# Potential Eligibility for Disease Modifying Therapies Among Individuals Referred to A Tertiary Care Memory Clinic in Calgary, Alberta, Canada: Implications for AD Biomarker Testing

**DOI:** 10.1002/alz.091118

**Published:** 2025-01-09

**Authors:** Gavin Thomas, Eric E. Smith, Zahinoor Ismail, Julia G Kirkham, Pamela Roach, Zahra Goodarzi, Mohammad Z I Chowdhury, Dallas Seitz

**Affiliations:** ^1^ University of Calgary, Calgary, AB Canada; ^2^ Hotchkiss Brain Institute, University of Calgary, Calgary, AB Canada

## Abstract

**Background:**

Potential disease modifying therapies (DMT) for Alzheimer’s disease (AD) may become available in Canada although it is unclear what percentage of people presenting to tertiary care memory programs may require beta‐amyloid testing be eligible for these treatments. We evaluated eligibility for potential DMTs among individuals in the Prospective Registry for Persons with Memory Symptoms (PROMPT) dementia research registry, comprising patients seen in a tertiary care cognitive clinic in Calgary, Alberta, Canada, based on cognitive criteria, medical history, and potential diagnosis of AD on clinical criteria.

**Method:**

We analyzed all individuals included in the PROMPT registry from July 2010 to May 2023 who were diagnosed with either mild cognitive impairment (MCI) or possible or probable dementia at their baseline assessment. AD biomarker testing is not routinely available in this clinic. The characteristics of PROMPT participants were then compared to the inclusion criteria used in recent trials of three DMTs. The proportions of individuals in the PROMPT registry who were potentially eligible for DMTs were then determined.

**Result:**

Of the 1,901 individuals in the PROMPT registry, 1,119 were diagnosed with MCI or dementia at their baseline visit (mean age 75 years and 54% male). Among the group with MCI or dementia, 86% (965) were diagnosed with either MCI or AD. Of the individuals diagnosed with MCI or AD, 55‐80% met cognitive test score eligibility for different DMTs at their initial clinic visit. Potential medical contraindications to treatment with DMTs were present in 27% of this sample. Of all individuals referred to the tertiary care cognitive disorders clinic diagnosed with MCI or AD, approximately 40‐58% could potentially be eligible for DMTs based on their initial clinic assessment. Of all individuals in the PROMPT dementia registry, between 20‐29% could potentially be candidates for DMTs pending neuroimaging and biomarker confirmation of eligibility.

**Conclusion:**

A high proportion of memory clinic referrals are diagnosed with MCI or AD and are potentially eligible for DMTs and would require beta‐amyloid biomarker testing for eligibility confirmation. This information will help better define resource requirements for implementing AD DMTs should these become available in Canada.

